# Relationship between Experimental Diet in Rats and Nonalcoholic Hepatic Disease: Review of Literature

**DOI:** 10.1155/2018/9023027

**Published:** 2018-10-31

**Authors:** Ayane A. Rodrigues, Raíssa S. B. Andrade, Daniel F. P. Vasconcelos

**Affiliations:** ^1^Postgraduate Program in Biomedical Sciences, Federal University of Piaui, Parnaiba, PI, Brazil; ^2^Postgraduate Program in Biotechnology, Federal University of Piaui, Parniba, PI, Brazil; ^3^Department of Biomedicine, Federal University of Piaui, Parnaiba, PI, Brazil

## Abstract

**Background:**

The pathophysiology of nonalcoholic fatty liver disease (NAFLD) is related to unhealthy lifestyles that combine sedentary lifestyle, hypercaloric diets, excessive saturated fats, refined carbohydrates, and high intake of fructose as a food additive to various processed products. Both the broader recognition of the disease and the additional efforts to elucidate the NAFLD pathogenesis have led to an increase in animal models in recent years*. Objective. *This review was performed to provide better understanding of the association between the NAFLD and animal models.

**Methods:**

The search in the literature occurred before May of 2018 in the PUBMED database.

**Results:**

Most studies investigating the influence of diet on liver fat content have been performed using a high-calorie diet that leads to a significant increase in fat content in the liver.

**Conclusion:**

The findings of this review show that diet is one of the factors that predisposes to the appearance of NAFLD and that the studies presented a wide variety of designs.

## 1. Introduction

The prevalence of predictive metabolic diseases, especially dyslipidemias and nonalcoholic fatty liver disease (NAFLD), has increased worldwide [1, 2]. NAFLD and metabolic diseases associated with obesity, diabetes mellitus, and dyslipidemia are a growing public health problem both in the United States and worldwide [3]. The global population is affected by NAFLD with rates between 25% and 30% with higher prevalence in the Middle East and South America. In North America and Europe, a quarter of the adult population is affected by NAFLD [4]. It is a leading cause of liver transplantation and, by 2030, it is predicted to become one of the most common reasons for transplantation in the United States [5].

This pathology has drawn attention in recent years. Diagnosis and assessment of NAFLD can be performed based on physical examination, history of the disease, ultrasound examination, and hepatic imaging [6]. Although other noninvasive tests have been suggested, they must still be validated in large scales [1]. NAFLD presents an episode of variable hepatopathy of simple steatosis to nonalcoholic hepatitis (NASH), which is localized in the presence of hepatitis and inflammation, related to the death of hepatocytes; many patients progress to insufficient fibrosis/cirrhosis and hepatic failure [7]. The NAFLD is diagnosed histologically when > 5% of hepatocytes/field present lipid accumulation. That is the gold standard mean of diagnosis and should be considered in patients with NAFLD who present high risk to have fibrosis and steatohepatitis [8, 9].

The pathophysiology of NAFLD is associated with unhealthy lifestyles, which combine sedentary lifestyle, hypercaloric diets, excessive saturated fat, refined carbohydrates, and high fructose intake as a food additive to various processed products [10, 11].

Although the etiologies of dyslipidemias and NAFLD are complex and multifactorial, these diseases contain an important genetic component that is influenced by environmental factors and life habits, especially the food consumption profile [2, 12].

Improved knowledge of the disease and its complementary data to elucidate the NAFLD pathogenesis have led to an increase in the animal models in recent years [13, 14].

Taking into account the increase in studies with NAFLD, the objective of this study was to investigate the different study designs that targeted the diet relationship with the appearance of NAFLD experimentally in rats.

## 2. Materials and Methods

This review was performed according to the PRISMA (Preferred Reporting Items for Systematic Reviews and Meta-Analyses) statement according to Moher et al., 2009 [15].

### 2.1. Eligibility Criteria

**Figure 1:**
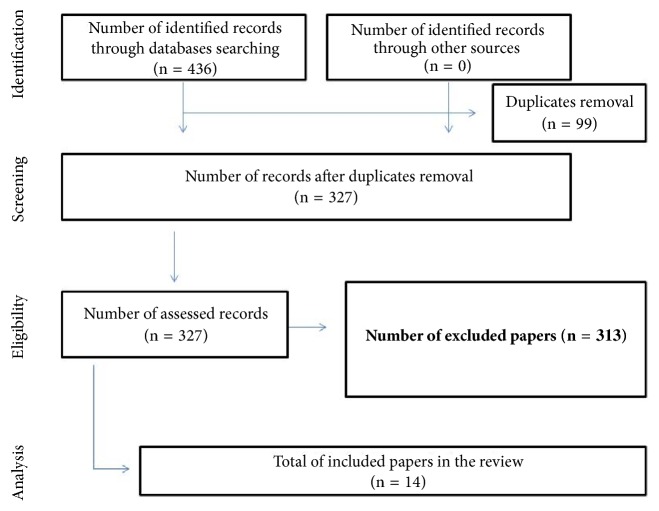
Flow diagram for identification, screening, eligibility, and analysis of studies included in the review.

For the eligibility of publications, each title and abstract was read exhaustively to confirm whether they addressed the guiding question of this research and whether it would meet the inclusion and exclusion criteria established. Then, the same happened to the stage of inclusion of the articles. The selection of studies is shown in Figure 1. In the identification and screening, the following inclusion criteria were adopted: be available on the electronic address, be free of charge, be fully presented, and be disclosed in English, Portuguese, or Spanish. Investigations that did not present enough information about NAFLD were excluded. In addition, dissertations, theses, reports, news, letters to the editor, and scientific articles not fully available online and those that were repeated in the databases were excluded. Studies that used diet inducing alcoholic liver disease (ALD) were also excluded.

### 2.2. Search Strategy

The following keywords or descriptors were used in the Health Sciences Descriptors (DeCS) of the Virtual Health Library: “NAFLD”, “diet”, and “rat model”.

The search in literature was executed by two investigators in the databases: Cochrane Library, Google Scholar, and PubMed for studies published before 28 Mai 2018 and addressing the NAFLD associated with rat model. The following combined keywords were used to search the literature: (“NAFLD”, “diet”, and “rat model”). Figure 1 demonstrates the search strategy and details from the review.

## 3. Results

Through the search carried out in PUBMED, 436 articles were found, of which 89 articles were not related to the theme, 8 articles were repeated, 2 articles were not available, 2 articles were excluded due to the used diet, which induces alcoholic liver disease (ALD) and thus was not the focus of this review, and 313 were related to the subject, but they were testing other compounds. This way, 14 articles were analyzed (Figure 1). Regarding the periodical and language, they were all published in international journals with English language.

As for the articles discussing diet and the onset of NAFLD, 1 used the iron-fructose-enriched diet (Ackerman et al., 2005) [15]; 2 induced steatohepatitis through the diet deficient in methionine-choline (Kirsch et al., 2003; Wu et al., 2011) [16, 21]; 1 used the high-fat diet associated with sucrose (Torres-Vilalobos et al., 2015) [23]; 1 used the high-fat, fructose, and high-fat diets associated with fructose (Lee et al., 2015) [24]; 2 used the high-fat diet (Zou et al., 2006; McDonald, 2011) [17, 20]; 2 used a high-fat and a methionine-deficient diet (Xu et al., 2010; De Lima, 2008) [18, 19]; 1 used rich diet and fat and diet deficient in methionine and choline (Han et al., 2017) [28]; 4 reviewed experimental models that induce NAFLD (Kucera, Cervinkova, 2014; Eslamparast et al., 2017; Goossens, 2017; Mikhail et al., 2017) [22, 25–27]. The respective data are presented in Table 1.

Dietary models are considered more similar to human metabolic diseases, but there are no standard composition and duration for these diets today: high-fat diets range from 30% to 60% fat content, including saturated fat, monounsaturated fatty acids, and polyunsaturated fatty acids, and last from a few days (short term) to more than a week (long term) [29]. In addition, theoretically composed diets of identical fat types can produce different results and data are often difficult to compare due to uncontrollable differences between primary fat sources and diet preparation [30].

It was observed that most articles found in the literature were related to the subject, but the studies used animal diet models to induce NAFLD in order to test compounds that interfere in the progression of the disease.

Ackerman et al. (2005) [15] demonstrated that the model of fructose-treated rats may be a suitable model for studying various aspects of human NAFLD, especially if it is associated with iron, since it generates an increase in liver fibrosis. This model contained 20.7% protein, 5% fat, 60% carbohydrate (fructose), 8% cellulose, 5% mineral blend, and 1% vitamin blend, combined with 50 mg of iron for each 1 kg diet during 5-week period. The model of fructose-iron showed evidence of mild to moderate deposition of macrovesicular and microvesicular fat with minimal signs of perisinusoidal fibrosis in 3 of 12 rats.

De Lima et al. (2008) [18] proposed a diet rich in fat (35% total fat, 54% transfatty acid) and choline-deficient for 16 weeks. This model demonstrated the development of histological NASH with cirrhosis, oval cell proliferation, and CK 19 positive hepatocellular carcinoma. In 2009, Xu et al. [19] tested a high-fat diet in rats and serum levels of glucose, triglyceride, cholesterol, alanine aminotransferase (ALT), free fatty acids (FFA), insulin, and tumor necrosis factor-alpha (TNF-alpha) were determined. Hepatic histology was also examined by H&E stain. The liver weight and liver index increased within one month, when hepatic steatosis was also observed. By month 2, the body weight and epididymal fat weight started increasing, which was associated with increased serum levels of FFA, cholesterol, and TNF-alpha, as well as development of fatty liver. The serum ALT level increased from month 3 on. Steatohepatitis occurred after three months. Thus, this model is recommended for the study of NASH and its implications.

The study by Wu et al. (2011) [21] and later Han et al. (2017) [28] used a diet deficient in choline/methionine (methionine 0.15%, choline 20 ppm, and 12% lipid component) for 2 weeks and concluded that researchers can directly measure the degree of steatosis with less concern about heterogeneous fatty infiltration and, moreover, provide a continuous, but not categorical, measure of hepatic steatosis.

The diet deficient in methionine and choline (MCD) in the study by Kirsch et al. (2003) [16] was composed of 2 g/kg of choline and 3 g/kg of methionine for 4 weeks. The research demonstrated profound differences between species, lineages, and sex in the nutritional model of MCD of NASH. From the groups studied, C57/BL6 male mice developed the histological characteristics that most resemble those observed in human NASH.

According to McDonald et al. (2011) [20], the high-calorie, high-fat diet with 41% fat energy was able to increase intra-abdominal fat, which preceded an increase in total body weight and was accompanied by metabolic abnormalities, including elevated triglyceride level and hyperglycemia in response to a glucose challenge.

**Figure 2:**
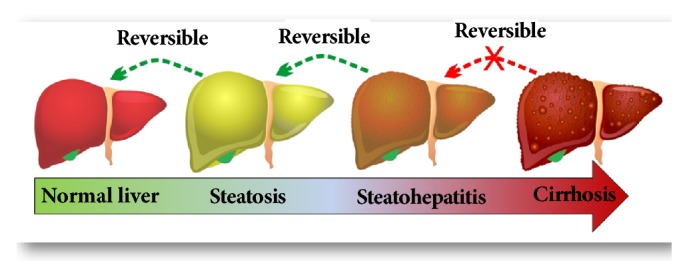
The progression of nonalcoholic fatty liver disease (NAFLD).

Zou et al. (2006) [17] also stated that the hypercaloric diet model (77% of fat energy, 14% of total milk powder, and 9% of carbohydrates) provides new opportunities to study the pathogenesis and treatment of the metabolic syndrome associated with steatohepatitis, such as obesity, abnormal aminotransferase, hyperlipidemia, hyperinsulinemia, hyperglycemia, and insulin resistance. The progression of NAFLD also must be highlighted, which has potential to advance to steatohepatitis or even cirrhosis, as seen in Figure 2. It was demonstrated that the hypercaloric diet models showed the same metabolic profile observed in humans, such as increased levels of insulin, insulin resistance, hyperglycemia, hyperleptinemia, glucose intolerance, and increased levels of visceral white adipose tissue [17, 31].

The study by Kucera and Cervinkova [22] aimed to review several diets used to develop NAFLD and discuss nutritional, genetic, and combined models of NAFLD, as well as pros and cons. The choice of a suitable animal model for this disease, while respecting its limitations, may help to understand its complex pathogenesis and discover new therapeutic strategies.

In 2015, Torres-Vilalobos et al. [23] aimed to evaluate the impact of four different diets on the production of NAFLD with emphasis on high combined consumption of high fat and sustained sucrose. Their methodology used the following diets: control diet, high-fat, and cholesterol + 5% sucrose diet in drinking water, high-fat corn starch diet + 5% sucrose in drinking water, and food diet + 20% sucrose in drinking water for 90 days. They concluded that the high-fat + cholesterol diet with combination of high fat and high sucrose is more effective in producing NAFLD compared with a high-sucrose diet only.

Lee et al. (2015) [24] observed consequences of high-fat (HF) and high-fructose (HFr) diets in rats. Intrahepatic inflammation and metabolic derangements were more prominent in the HF and HFr combination model than in the HF monodiet model, which shows that a type of sugar aggravates the liver disease when associated with fat.

Eslamparast et al. (2017) [26] proposed in a review that poor dietary composition is an important factor in the progression of NAFLD and proposed that “high-quality healthy diet” improves hepatic steatosis and metabolic dysfunction in patients with NAFLD. They highlighted that most patients with NAFLD follow diets with overconsumption of simple carbohydrates and total and saturated fat, with reduced intake of dietary fiber and omega 3-rich foods.

Studies investigating the influence of diet on liver fat content were performed using a high-calorie diet that leads to a significant increase in liver fat content. DHC is well known to induce some metabolic disorders, and the consequences are completely dependent on the composition and duration of the diet [32, 33].

A broad view of the publications that study the diets used to simulate NAFLD in animals shows a break in nutritional, genetic, and combined diets. Goossens and Jornayvaz [27] describe the nutritional models of NAFLD that tried to mimic the metabolic disorders observed in the disease, as well as the histological alterations in the liver: hyperlipid diet, diet deficient in methionine and choline, high-cholesterol diet, high-fructose diet, ketogenic diet, and other models. Among these diets, Goossens and Jornayvaz [27] described the fat-rich diet model mentioned, tested in male Sprague-Dawley rats by Lieber et al. [34] (71% fat energy, 11% carbohydrate, and 18% protein). They demonstrated the development of steatosis in three weeks associated with insulin resistance and increased fibrogenesis markers. This same study also suggests that the fructose-rich diet model associated or not with the high-fat model induces steatosis over a period of four to eight weeks. DHC is well known to induce some metabolic disorders and the consequences are completely dependent on the composition and duration of the diet [29].

According to Mikhail et al. [25], the chemical models are divided into streptozotocin, carbon tetrachloride, and diethylnitrosamine, while the genetic models are models of diabetes mellitus type 2 and models of atherosclerosis and hepatocellular carcinoma.

These models allow researchers to control,* in vivo*, genetic and environmental factors that may influence the development of the disease and its secondary complications [29], thus obtaining useful information about its management and treatment in humans.

## 4. Conclusion

In conclusion, the findings of this review show that the diet is one of the factors that predisposes to the appearance of NAFLD and that the studies presented a wide variety of designs, with the DHC being the most frequent diets in studies with experimental models of NAFLD. These reinforce that animal diet models are able to mimic the pathophysiological characteristics of NAFLD and are still widely used in research, mainly related to the testing of compounds that interfere with the progression of the disease.

## Figures and Tables

**Table 1:** Characteristics of the studies included in this study.

Authors and year of publication	Title of the article	Objective	Diet model	Results
Kirsch et al., 2003 [16]	Nutritional model of non-alcoholic steatohepatitis: species studies, strains and sex differences	To investigate the differences between species, lineages, and sex in the nutritional model of diet deficient in methionine choline to induce nonalcoholic steatohepatitis (NASH).	They were fed with a diet deficient in methionine and choline; the control group received an identical diet to which choline bitartrate (2 g / kg) and DL-methionine (3 g / kg) were added.	The present study has demonstrated profound species, strain, and sex differences in the MCD nutritional model of NASH.

Ackerman et al., 2005 [15]	Hepatic Effects of Blood Pressure and Reduction of Plasma Triglycerides	To characterize hepatic pathology and function, hepatic lipid composition and hepatic iron concentration, and fasting plasma insulin changes occurring in rats as a result of the fructose-enriched diet, with and without therapeutic maneuvers to reduce blood pressure and plasma triglycerides.	Diet enriched with fructose (as supplied by Harlan Teklad) 20.7% (by weight basis) of protein (as casein), 5% fat (as lard), 60% carbohydrates (as fructose), 8% cellulose, 5% mineral blend, and 1% blend of vitamins. This diet contains 50 mg of iron in 1 kg of diet. For 5 weeks.	This study demonstrates that the model of fructose-treated rats can be a suitable model for studying various aspects of human NAFLD.

Zou. et al., 2006 [17]	High-fat emulsion-induced rat model of non-alcoholic steatohepatitis	The objective of the present study is to produce a practical and repetitive experimental rat model for steatohepatitis by designing a high-fat emulsion containing high fat, sucrose, and protein.	The rats were divided into normal control group (NC group) and hyperlipid emulsion model group (HF group). All rats received standard ration and water. In addition, they had free access to a sucrose solution (18%). Model mice were orally treated with the high-fat emulsion (10 ml / kg) once daily. For 6 weeks.	A new rat model of steatohepatitis was established. This model provides new opportunities to study the pathogenesis and treatment.

De Lima et al., 2008 [18]	A model of NASH rodents with cirrhosis, oval cell proliferation and hepatocellular carcinoma	Produce a mouse model of NASH, cirrhosis, and HCC.	Diet rich in fat (35% total fat, 54% trans fatty acid) and choline-deficient for 16 weeks	Our initial experience with this model demonstrates the development of histological NASH with cirrhosis, oval cell proliferation, and CK 19 positive hepatocellular carcinoma.

Xu et al., 2009 [19]	Characterization of High-Fat, Diet-Induced, Non-alcoholic Steatohepatitis with Fibrosis in Rats	Create a new NASH model by feeding animals on a high-fat diet (DHC), with a hope of reproduction as key features of human NASH for future research.	DHC was composed of the following energy sources: 52% were provided by carbohydrates, 30% by fat, and 18% by protein (total calories: 4.8 kcal/g). Comparison with the diet deficient in methionine-choline.	In conclusion, we created a practically simple but accurate rat NASH model. It reproduced the pathological sequence of events typical of human NASH.

McDonald et al., 2011 [20]	Adverse metabolic effects of a high-calorie, high-fat diet (HFD) in rodents precede observable changes in body weight	The specific objective was to determine the impact of a lifetime exposure to a hypercaloric, DH on adiposity, its distribution, and its glycemic control markers	Hyperlipid diet with 5% fat energy in the control group and a hypercaloric diet, rich in 41% fat, for 39 weeks.	In conclusion, after only 4 weeks, animals fed on a HFD from weaning had increased intra-abdominal fat, which preceded an increase in overall body weight and was accompanied by metabolic abnormalities including high TG and hyperglycemia in response to a glucose challenge.

Wu et al., 2011[21]	Hepatic steatosis: an experimental model for quantification	Introduce an experimental method to quantify hepatic steatosis in rats and provide an alternative experimental model in the following studies.	Two groups fed with commercial diet and choline/methionine deficient-diet (DMC) for two weeks.	By this model, researchers can directly measure the degree of steatosis with a lesser concern about heterogeneous fatty infiltration and provide a continuous but not categorical measurement of hepatic steatosis.

Kucera, Cervinkova, 2014 [22]	Experimental models of non-alcoholic fatty liver disease in rats	This article reviews the widely used experimental models of NAFLD in rats. We discuss the nutritional, genetic, and combined models of NAFLD and its pros and cons.	Diets high in fat; diet rich in fructose/sucrose; diet deficient in methionine and choline; atherogenic diets; genetic and combined models.	Although there is no ideal model for human nonalcoholic fatty liver disease that reflects all the clinical aspects of human disease, choosing an appropriate model for studying particular events of NAFLD while respecting its limitations has contributed greatly to the understanding of this disease and its progression and treatment.

Torres-Vilalobos G, et al., 2015[23]	High-fat combined diet and sustained high sucrose consumption promotes NAFLD	To evaluate the impact of 4 different diets on the production of NAFLD with emphasis on a high combined consumption of high fat and sustained sucrose.	(1) Control diet. (2) High fat and cholesterol + 5% sucrose diet in drinking water. (3) High-fat corn starch diet + 5% sucrose in drinking water. (4) Food diet + 20% sucrose in drinking water for 90 days.	The HFC diet with combination of high fat and high sucrose is more effective in producing NAFLD compared with a high-sucrose diet only.

Lee et al., 2015 [24]	Histological and Metabolic Breakdown in high-fat, high-fructose and combined dietary animal models	To evaluate and compare the differences in the induction of histological and metabolic characteristics induced by the combined diets to characterize the resulting NAFLD and NASH mouse models.	G1: normal ration, G2: hyperlipid diet fed on high-fat diet (60% total calories), G3: high fructose (30% fructose in drinking water), G4: high fat + high fructose combination (60% fat + 20% fructose) for 20 weeks.	Intrahepatic inflammation and metabolic derangements were more prominent in the HF and HFr combination model than in the HF monodiet model.

Mikhail et al., 2017 [25]	Animal Models of Non-Alcoholic Fatty Liver Disease - A Beginner's Guide	This review provides a brief overview of the most commonly used animal models in NDFL research and maternal health care, following the broader maritime guidance model, focusing on the key phenotypic characteristics of each model.	Diet deficient in methionine and choline; diet deficient in hill-deficient L-amino acids; atherogenic diet; fructose; high-fat diet, variations in high-fat diet.	This review presents a brief overview of these models with a particular focus on the basic mechanisms and physical, biochemical, and histological phenotypes.

Eslamparast et al., 2017 [26]	Independent dietary weight loss composition in the treatment of non-alcoholic fatty liver disease	Literature review evaluating the evidence behind dietary components, including meat, omega-3-rich diets, and depending on more evidence, we agree with the EASL-EASO Clinical Guidelines recommendation of the Mediterranean diet as the diet of choice in these patients.	Dietary Approach to Stop Hypertension (DASH) is a plant-rich dietary standard, Fiber Intervention (Soluble, Prebiotic), Omega-3 Intervention, Low Fat Interventions vs. Low-CHO, probiotics.	At this time, we do not have a definitive answer for the optimal proportions of total macronutrients (CHO, fat, and protein) in the diet of NAFLD patients.

Goossens, Jornayvaz, 2017 [27]	Translational aspects of diet and non-alcoholic fatty liver disease	Discuss some key questions of the translational aspects of NAFLD research from the perspective of nutrition and dietary interventions, literature review.	High-fat, methionine-deficient diet; high cholesterol; high fructose and ketogenic content.	NAFLD is a complex disease with an increasing epidemiology. Currently, no specific therapeutic alternative has been developed, partly due to the lack of robust reproducible dietary animal models of the disease.

Han et al., 2017 [28]	Distinction of the Metabolic Profile of the Progression of Non-Alcoholic Fatty Liver Disease from a Common Mouse Model	Perform complete metabolomic analyses on liver samples to determine which pathways are more markedly altered in the human condition and compare these changes with nonalcoholic fatty liver (NAFLD) models.	Alternately, the rats were fed on high-fat diet (high cholesterol, 18% butterfat) or methionine-choline deficient diet for eight weeks.	These results indicate that metabolites of specific pathways may be useful biomarkers for NASH progression, although these markers may not correspond to rodents.

## Data Availability

The data used to support the findings of this study are included within the article.
